# Radiomics from Routine CT and PET/CT Imaging in Laryngeal Squamous Cell Carcinoma: A Systematic Review with Radiomics Quality Score Assessment

**DOI:** 10.3390/cancers18020237

**Published:** 2026-01-13

**Authors:** Amar Rajgor, Terrenjit Gill, Eric Aboagye, Aileen Mill, Stephen Rushton, Boguslaw Obara, David Winston Hamilton

**Affiliations:** 1 Population Health Sciences Institute, Faculty of Medical Sciences, Newcastle University, Newcastle-Upon-Tyne NE1 7RU, UK; david.hamilton@ncl.ac.uk; 2 National Institute for Health & Care Research, Newcastle University, Newcastle-Upon-Tyne NE1 7RU, UK; 3 Newcastle-Upon-Tyne Hospitals NHS Foundation Trust, Freeman Hospital, Freeman Road, Newcastle-Upon-Tyne NE7 7DN, UK; 4 Faculty of Life Sciences and Medicine, King’s College London, London SE1 1UL, UK; terrenjit.gill@kcl.ac.uk; 5 Imperial College London Cancer Imaging Centre, Department of Surgery & Cancer, Hammersmith Hospital, London W12 0HS, UK; eric.aboagye@imperial.ac.uk; 6 Modelling, Evidence and Policy, School of Natural and Environmental Sciences, Newcastle University, Newcastle-Upon-Tyne NE1 7RU, UK; aileen.mill@newcastle.ac.uk (A.M.); steven.rushton@newcastle.ac.uk (S.R.); 7 School of Computing, Newcastle University, Newcastle-Upon-Tyne NE1 7RU, UK; boguslaw.obara@ncl.ac.uk

**Keywords:** radiomics, laryngeal cancer, larynx, head and neck cancer, biomarkers, artificial intelligence, treatment outcome

## Abstract

**Background/Objectives**: Radiomics, the high-throughput extraction of quantitative features from medical imaging, offers a promising method for identifying laryngeal cancer imaging biomarkers. We aim to systematically review the literature on radiomics in laryngeal squamous cell carcinoma, assessing applications in tumour staging, prognosis, recurrence prediction, and treatment response evaluation. PROSPERO ID: CRD420251117983. **Methods**: MEDLINE and EMBASE databases were searched in May 2025. Inclusion criteria: studies published between 1 January 2010 and 31 January 2024, extracted radiomic features from CT, PET/CT, or MRI, and analysed outcomes related to diagnosis, staging, survival, recurrence, or treatment response in laryngeal cancer. Exclusion criteria: case reports, abstracts, editorials, reviews, or conference proceedings, exclusive focus on preclinical or animal models, lack of a clear radiomics methodology, or did not include imaging-based feature extraction. Results were synthesised narratively by modelling objective, alongside formal assessment of methodological quality using the Radiomics Quality Score (RQS). **Results**: Twenty studies met the inclusion criteria, with most using CT-based radiomics. Seven incorporated PET/CT. Radiomic models demonstrated moderate-to-high accuracy across tasks including T-staging, thyroid cartilage invasion, survival prediction, and local failure. Key predictive features included first-order entropy, skewness, and texture metrics such as size zone non-uniformity and GLCM correlation. Methodological variability, limited external validation, and small samples were frequent limitations. **Conclusions**: Radiomics holds strong promise as a non-invasive biomarker for laryngeal cancer. However, methodological heterogeneity identified through formal quality assessment indicates that improved standardisation, reproducibility, and multicentre validation are required before widespread clinical implementation.

## Simple Summary

This review presents a timely and thorough synthesis of the rapidly evolving field of radiomics as applied to laryngeal squamous cell carcinoma. Radiomics holds real potential as a powerful non-invasive tool for extracting quantitative imaging biomarkers from routine CT and PET/CT scans. These biomarkers offer novel opportunities to improve tumour staging, risk stratification, prognosis, recurrence prediction, and treatment response assessment in laryngeal cancer—areas where current clinical tools are limited. Our analysis of 20 relevant studies reveals consistent radiomic features such as entropy, skewness, and texture-based metrics that demonstrate promising prognostic value across multiple clinical outcomes. We also undertake a formal assessment of methodological quality using the Radiomics Quality Score (RQS). This assessment highlights substantial variability across studies, reflecting common challenges including small sample sizes, heterogeneous patient cohorts, and insufficient external validation. Together, these findings indicate that while radiomics shows promise in laryngeal cancer, improved standardisation, reproducibility, and validation are required before widespread clinical implementation.

## 1. Introduction

**Figure 1:**
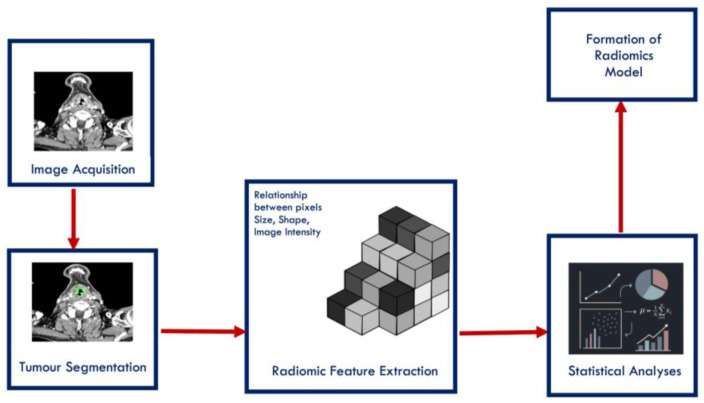
Formation of a radiomics model workflow diagram.

Laryngeal cancer represents a complex clinical entity, particularly in its advanced stages, where curative treatment options often carry significant trade-offs in terms of function and quality of life. In recent years, radiomics has emerged as a promising avenue for individualised care. Radiomics refers to the extraction of quantitative data from medical imaging, including computed tomography (CT), magnetic resonance imaging (MRI), and positron emission tomography (PET). These features can describe tumour morphology, texture, and intensity patterns that may reflect underlying histological or molecular characteristics of the disease [1]. When incorporated into predictive or prognostic models, often alongside clinical, demographic or genomic variables, radiomic features can serve as imaging biomarkers to support more individualised care. Figure 1 illustrates the standard radiomics pipeline and the process by which a model is developed.

Radiomics offers the potential to act as a ‘virtual biopsy’, providing non-invasive, repeatable assessments of the whole tumour and its microenvironment [2]. It may also assist in stratifying patients by risk of recurrence, resistance to chemoradiotherapy (CRT), or early treatment failure, areas where conventional assessment tools have shown limited predictive accuracy.

There is increasing availability of open source radiomics software, consensus guidelines on image standardisation, and growing interest in artificial intelligence applications in oncology. As clinical trials and multi-institutional datasets become more accessible, it is timely to consolidate current findings, assess reproducibility, and identify translational pathways to support integration into clinical workflows.

Despite this progress, the radiomics evidence base specific to laryngeal squamous cell carcinoma remains relatively limited with many studies aggregating multiple head and neck subsites despite known differences in tumour biology, treatment paradigms, and imaging phenotypes. A laryngeal-specific synthesis is therefore required to avoid inappropriate generalisation and to critically evaluate the strength and limitations of the available evidence. Moreover, although radiomics has been explored across a range of imaging modalities, this review deliberately focuses on radiomic analyses derived from routine CT and PET/CT imaging, which form the backbone of contemporary laryngeal cancer management. Imaging techniques such as endoscopic or optical approaches, which rely on fundamentally different acquisition paradigms and analytical frameworks, were therefore considered outside the scope of this radiomics-focused synthesis.

Accordingly, this review aims to evaluate the quality, consistency, and clinical relevance of the existing evidence, identify key methodological and biological patterns across studies, and critically appraise the limitations that currently constrain translation of radiomics into routine clinical practice for laryngeal cancer [3].

## 2. Materials and Methods

### 2.1. Data Sources and Searches

This study was registered on PROSPERO (ID: CRD420251117983). The PRISMA guideline for reporting of systematic reviews [4] was used to perform a systematic literature search in the electronic biomedical databases of MEDLINE and EMBASE using the Ovid interface. The search strategy was designed to identify original research studies that evaluated radiomic features extracted from clinical imaging in relation to laryngeal cancer outcomes. Boolean logic was used to combine disease-related keywords (“laryngeal cancer” OR “larynx” OR “larynx cancer”) with informatics-related terms (“radiomics” OR “texture analysis” OR “machine learning” OR “artificial intelligence”). The full electronic search strategy is provided in Supplementary S1.

The search was limited to English-language articles published between 1 January 2010 and 31 January 2024. This window was chosen to reflect the timeframe during which radiomics has become increasingly adopted within oncological research, whilst allowing breadth for the inclusion of early exploratory studies. Duplicate entries were removed, and additional eligible articles were identified through backward citation-tracking of included papers and relevant systematic reviews. Ethical approval was not required for this review.

### 2.2. Study Selection

Eligible studies met the following criteria if they, as follows:

1. Included human patients diagnosed with laryngeal squamous cell carcinoma (LSCC) (or a clearly defined subsite within a broader head and neck cohort).
2. Employed radiomic feature extraction from CT, PET/CT, or MRI scans.
3. Investigated outcomes related to diagnosis, staging, survival, recurrence, treatment response, or prognosis.
4. Used a defined and reproducible image analysis pipeline.
5. Provided statistical evaluation of radiomic model performance (e.g., AUC, concordance index, and accuracy).
6. Were original, peer-reviewed full-text articles published in English.

Exclusion criteria included case reports, abstracts, editorials, reviews, or conference proceedings, exclusive focus on preclinical or animal models, a lack of a clear radiomics methodology, or if they did not include imaging-based feature extraction.

### 2.3. Data Extraction and Risk of Bias Assessment

**Figure 2:**
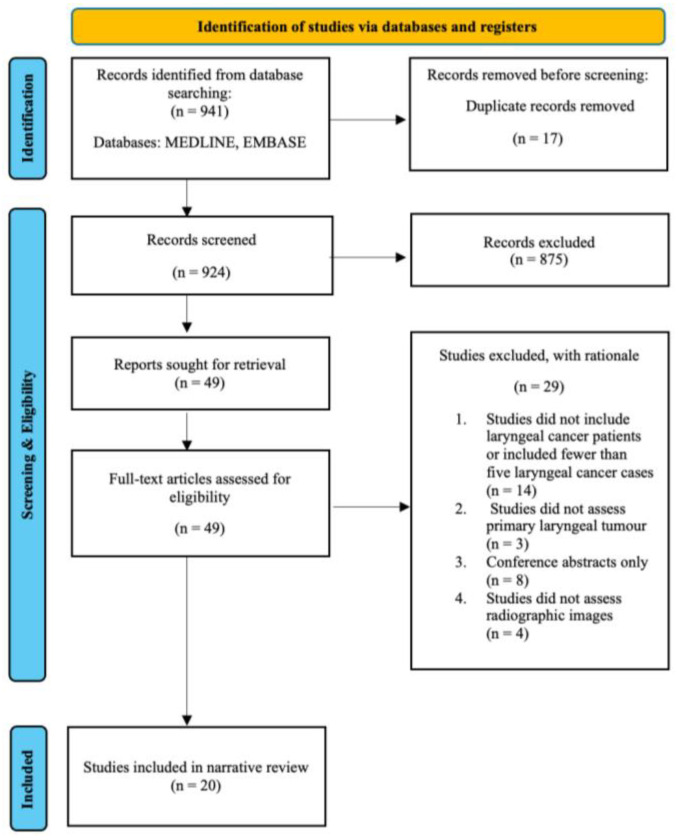
PRISMA flow diagram of study selection.

Following database searching, all records were imported into EndNote for deduplication. Two reviewers independently screened titles and abstracts for relevance. Full texts were then retrieved for all potentially eligible articles, and inclusion was confirmed based on predefined criteria. Discrepancies were resolved by consensus. The screening and selection process is illustrated in Figure 2.

A structured data extraction template was developed, incorporating key study attributes including author and year, radiomics software used, imaging modality, study aim and design, patient cohort characteristics, modelling strategy, statistical methods, significant radiomic features, and outcome metrics. Where relevant, the presence of external validation, compliance with Image Biomarker Standardisation Initiative (IBSI) recommendations [5], and model interpretability were also recorded.

### 2.4. Radiomics Quality Score Assessment

To support critical appraisal as part of the review design, formal methodological quality assessment was undertaken using the Radiomics Quality Score (RQS) [1]. Each included study was evaluated across predefined RQS domains encompassing imaging protocol reporting, feature robustness, validation strategy, statistical analysis, clinical utility, and transparency. Scoring was performed conservatively based on explicit reporting within each publication, with unreported criteria scored as zero; where applicable, negative scores were assigned in accordance with the RQS framework. Two reviewers independently performed the quality assessment, with discrepancies resolved by consensus. In addition to formal scoring, narrative synthesis was used where appropriate to provide contextual interpretation of study robustness, heterogeneity, translational relevance, and methodological limitations across the body of evidence.

## 3. Results

### 3.1. Study Characteristics

A total of 20 eligible studies were included in this review following full-text screening. They varied in imaging modality, radiomics software, modelling objective, sample size, and validation strategy. The most common imaging modality was CT, used in 13 studies. Seven studies used 18F-FDG PET/CT, either as the sole modality or alongside CT-derived radiomics. One study incorporated CT perfusion imaging alongside conventional radiomics. Six studies focused exclusively on laryngeal cancer cohorts, only three of which examined prognostic outcomes as their primary objective.

Given the heterogeneity of study designs, populations, and imaging pipelines, the studies were synthesised according to their primary modelling objective. This approach was selected to ensure alignment with the translational focus of this review and to enable meaningful comparison across imaging techniques. The four categories used for synthesis were as follows:

1. Tumour staging and histological grading.
2. Survival prediction (overall and disease-specific).
3. Recurrence and progression modelling.
4. Treatment response prediction, including CRT failure.

**Figure 3:**
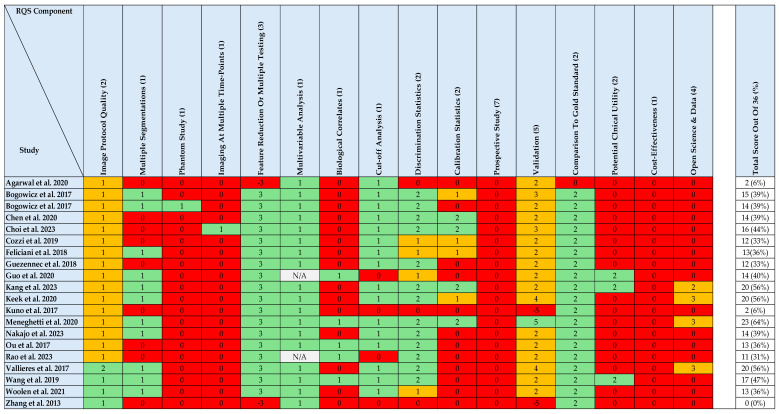
Methodological quality assessment of included studies [6,7,8,9,10,11,12,13,14,15,16,17,18,19,20,21,22,23,24,25] using the Radiomics Quality Score. Individual RQS components are displayed for each study, with colour coding indicating criteria met (green), criteria partially met or not fully reported (orange), and criteria not met or subject to negative scoring where methodological penalties were applied (red); grey denotes criteria that were not applicable. Numbers in parentheses indicate the maximum possible score for each RQS component. Total RQS scores are presented as a percentage of the maximum possible score, illustrating variability in methodological quality across the literature.

Studies that addressed more than one endpoint were categorised according to their primary outcome of interest. Table 1 and Table 2 are intended as detailed reference summaries, providing consolidated methodological and clinical information from each included study to support transparency and enable comparison across radiomics pipelines, imaging modalities, and outcomes. In addition to descriptive study-level reporting, the synthesis prioritised cross-study aggregation of recurring radiomic feature classes, evaluation practices (e.g., discrimination, calibration, and validation), and methodological limitations affecting reproducibility and generalisability. The methodological quality of each study was formally assessed using the RQS and is reported in Figure 3.

### 3.2. Radiomics for Tumour Staging and Histopathological Grading

Wang et al. sought to classify T3- versus T4-stage tumours in a cohort of 211 patients with advanced laryngeal SCC. Using contrast-enhanced CT and Pyradiomics, they extracted a broad range of first-order, shape and wavelet-transformed features. Following feature selection with least absolute shrinkage and selection operator (LASSO), which is a statistical approach that prevents overfitting, a support vector machine (SVM) model was developed. The radiomics-based classifier demonstrated excellent predictive accuracy (AUC of 0.892 in the validation cohort) [6]. Combining radiomics with radiologist interpretation improved performance further, highlighting the potential for these tools to complement clinical expertise. However, the study’s retrospective design and lack of external validation limit its generalisability.

Guo et al. investigated the prediction of thyroid cartilage invasion, a key criterion for defining T4a disease. In a cohort of 236 patients, they developed logistic regression models using radiomic features extracted from contrast-enhanced CT, implementing class-balancing and rigorous cross-validation. Their model achieved an AUC of 0.905, significantly outperforming junior radiologist assessments [7]. This reinforces the value of quantitative imaging for detecting subtle anatomical invasion that may be missed on routine interpretation. As with other work in this space, variability in scanner types and lack of external validation remain important considerations.

Rao et al. conducted an exploratory study evaluating the role of radiomics in T-stage classification and histopathological tumour grading. Their analysis, restricted to 20 supraglottic laryngeal tumours, applied a minimum redundancy–maximum relevance approach followed by SVM modelling. Although limited by sample size, the study demonstrated moderate discriminatory ability (AUCs close to 0.79 for staging and 0.69 for grade classification) [8]. These findings suggest potential for radiomics to reflect underlying tumour biology, but also underscore the need for validation in larger, well-characterised cohorts.

### 3.3. Radiomics for Survival Prediction and Recurrence

A total of 14 studies identified in this review addressed prognostic modelling, incorporating endpoints such as overall survival (OS), progression-free survival (PFS), disease-free survival (DFS), and locoregional recurrence (LRR). Imaging modalities included contrast-enhanced CT and 18F-FDG PET/CT, with most studies using retrospective designs and internal validation. The studies are summarised in Table 2 and discussed below. Across these studies, reported prognostic performance was frequently derived from retrospective cohorts with internal validation only, limiting the robustness and generalisability of many proposed models despite apparently strong discrimination metrics.

The earliest prognostic investigation amongst the included studies was conducted by Zhang et al., who analysed CT-based first-order features in 72 head and neck SCC patients (21 laryngeal). Texture entropy and skewness were found to be independently associated with OS, with multivariate hazard ratios (HR) of 2.10 and 3.67, respectively [9]. Whilst promising, the study lacked external validation and was limited by its heterogeneous population.

Ou et al. similarly developed a CT-based radiomics signature in 120 patients (18 laryngeal), incorporating 24 radiomic features with principal component analysis-based dimensionality reduction. Their model predicted both OS and PFS with 5-year AUCs of 0.78 when combined with p16 status, demonstrating added value over clinical variables alone [10]. Notably, patients with high radiomic risk scores showed significantly better outcomes with CRT compared to bioradiotherapy, suggesting a role for treatment stratification.

Bogowicz et al. evaluated radiomic features from both PET and CT scans. Their combined PET-CT model achieved a C-index of 0.77 for local control in training and 0.73 in validation. Grey-level size zone matrix (GLSZM)-derived features such as size zone entropy (CT) and small zone low grey level emphasis (PET) emerged as significant predictors [11]. The study was notable for directly comparing imaging modalities and highlighting complementary prognostic value.

PET-based prognostic work was expanded by Feliciani et al. and Guezennec et al. The former used an open-source package “CGITA” and extracted features in 90 patients (14 laryngeal), identifying grey-level run-length matrix (GLRLM) low intensity long run emphasis (LILRE) as an independent predictor of local failure, with a C-index of 0.76 for the radiomics model versus 0.65 for clinical features alone [12]. Guezennec et al., in a larger cohort of 284 patients (32 laryngeal), showed that metabolic tumour volume (MTV) and grey-level co-occurrence matrix (GLCM) correlation were independently associated with OS, with multivariate HRs of 2.01 and 4.51, respectively. AUCs for MTV and GLCM correlation were 0.68 and 0.66, respectively, highlighting moderate prognostic value [13].

A multicentre study by Vallières et al. validated PET-CT radiomics signatures for multiple endpoints including OS, LRR, and distant metastasis. GLSZM and GLRLM features were repeatedly identified across endpoints, reinforcing their prognostic relevance. However, the cohort included only 45 laryngeal cancer patients [14].

More recent studies have applied delta-radiomics and hybrid modelling approaches. Choi et al. used paired pre- and post-treatment PET/CT scans to compute delta-radiomic scores (Rad-scores) in 91 patients (57 laryngeal). Their combined clinical–radiomic model achieved a C-index of 0.958 for OS and 0.889 for PFS. High prognostic weight was attributed to SUV variance, co-occurrence metrics, and skewness-related features [15].

Nakajo et al. focused exclusively on 49 laryngeal cancer patients, combining PET-derived GLCM entropy and GLZLM zone length non-uniformity (ZLNU) with clinical features in a random survival forest model. Their model achieved strong performance (C-indices of 0.808–0.840 in internal validation) [16]. Similarly, Kang et al. created a CT radiomics-enhanced nomogram for advanced laryngeal cancer, achieving validation AUCs of 0.735 (1-year OS) and 0.746 (3-year OS), with important features including GLSZM size zone non-uniformity and neighbouring grey tone difference matrix (NGTDM) complexity [17].

Among studies incorporating both radiomics and perfusion imaging, Woolen et al. evaluated delta radiomic features from paired pre- and post-treatment scans to predict one-year disease-free survival. While overall discrimination was modest (validation AUC = 0.69), the imaging-based model demonstrated greater prognostic discrimination than laryngoscopic assessment of treatment response alone (AUC = 0.40). Although laryngoscopy is not used in isolation for prognostication, it remains essential for direct visual assessment and clinical decision-making following definitive treatment. These findings therefore support a complementary role for quantitative imaging biomarkers alongside standard evaluation [18].

Some studies also examined local control or laryngectomy-free survival. Agarwal et al. used medium-filtered CT texture features such as entropy and kurtosis to predict these outcomes, reporting entropy as an independent predictor with *p* < 0.001 [19]. Meneghetti et al. performed external validation of their radiomics signature in 85 patients, reporting moderate C-indices of 0.66 for local control. Despite low laryngeal numbers, this study offered one of the few multicentre validations [20].

### 3.4. Radiomics for Treatment Response Prediction and Failure

Bogowicz et al. developed a model using post-treatment PET/CT radiomics to predict local tumour control. Although the cohort was heterogeneous (11 laryngeal), the radiomics models based on histogram range and GLCM difference entropy achieved promising C-indices of 0.71–0.73 in validation, suggesting meaningful signal even after treatment. A second study by the same group, combining CT and PET radiomics pre-treatment, reported comparable performance (C-index of 0.73 for the combined model), again highlighting the value of GLSZM-derived features such as small zone low grey level emphasis [11].

Feliciani et al. evaluated whether pre-treatment PET radiomics could predict local failure after CRT. Their model, incorporating GLRLM LILRE, outperformed clinical models (C-index of 0.76 versus 0.65) and identified LILRE as an independent predictor in multivariate analysis. Although there were 14 laryngeal cancer patients, the study underscored the potential of radiomics for early failure prediction in a CRT context [12].

Similarly, Choi et al. explored the use of delta-radiomic features derived from pre- and post-treatment PET/CT scans. In a mixed cohort including 57 laryngeal cancer patients, they developed Rad-scores that were significantly associated with both PFS and OS. Their combined clinical radiomic model achieved very high C-indices (0.889 for PFS and 0.958 for OS) [15]. Although the primary endpoints were survival-based, the use of treatment-induced radiomic change aligns this study more closely with treatment response modelling.

Nakajo et al. focused exclusively on 49 laryngeal cancer patients. They developed a model predicting both disease progression and PFS. Radiomic features such as GLCM entropy and GLZLM ZLNU were consistently selected, and the resulting model achieved strong performance with AUCs and C-indices exceeding 0.80. These results provide encouraging evidence that PET-based radiomics can identify patients at higher risk of progression early in their treatment pathway [16].

**Table 1:** Summary of CT-based radiomic studies for tumour staging and histopathological grading in laryngeal cancer.

Author, Year, and Theme	Study Aim	Study Type and Design	Imaging Modality	Radiomics Software Used	Exclusively Laryngeal Cancer Patients	Total Patients (n)	Laryngeal Cancer Patients (n)	Primary Treatment	Model Validation Strategy	Radiomics Feature Selection and Signature Construction	Significant Radiomic Features	Model Performance/ Statistical Outcomes	Limitations	Conclusions
**CT Radiomics for Staging and Grading in Laryngeal Cancer**														
**Wang et al. [6]** **2019** **Staging**	To determine whether CT radiomics can improve T-stage classification (T3 vs. T4) in advanced laryngeal cancer.	Retrospective single-institution cohort with internal validation using a train–test division.	Contrast-enhanced CT	Pyradiomics	Yes	211	n = 211 (T1–2: 0, T3–4: 211)	Surgery ± adjuvant therapy	Internal validation: 70/30 train–test split (n = 150/61)	**Feature selection**: ICC filtering (threshold ≥ 0.75) to retain stable features; LASSO used to reduce dimensionality and identify significant predictors **Signature construction:** Selected features used to train an SVM model; model parameters optimised via grid search and cross-validation; radiomics signature derived from trained SVM	**T Stage Classification (T3 vs. T4)**: First-order: Skewness, 2D mean intensity Shape: Least axis length, Sphericity Wavelet (LLH): Kurtosis (first-order), IDN (GLCM), Median (first-order) Wavelet (LLL): IMC (GLCM)	**T-stage Classification (Nomogram + Radiologist Assessment)**: AUC (Training): 0.899 (95% CI: 0.850–0.947) AUC (Validation): 0.892 (95% CI: 0.811–0.974)	Retrospective, single centre study. No external validation performed.	Integrating CT radiomics with radiologist assessment significantly improved T-stage classification accuracy in advanced laryngeal cancer, demonstrating strong predictive performance in both training and validation cohorts.
**Guo et al. [7]** **2020** **Staging**	To evaluate the potential of CT-based radiomic features in predicting thyroid cartilage invasion in laryngeal cancer.	Retrospective single-institution cohort with internal validation using cross-validation.	Contrast-enhanced CT	Radcloud and Anaconda3 (Python 3.6-based)	Yes	236	n = 236 (T1–2: 0 T3–4: 236)	Not applicable (Diagnostic)	Internal validation: 5-fold cross-validation	**Feature selection**: Standardisation followed by Kruskal–Wallis test to exclude scanner-dependent features; LASSO with 10-fold CV applied; features retained if LASSO coefficients > 0.04; SVMSMOTE used to address class imbalance **Signature construction**: Logistic regression models built on LASSO-selected and LASSO + SVMSMOTE feature sets; evaluated using 5-fold cross-validation	**Distinguishing Thyroid Cartilage Invasion**: Shape: LeastAxis, Elongation, Flatness FO: Kurtosis (logarithm, LLH, LLL), 10Percentile (square), Skewness (LHL, HHL), Energy (HLL) GLCM: Imc1 (exponential), ClusterShade (LHH), ClusterProminence (LLH), Correlation (LLL) GLRLM: HGLRE (logarithm), SRHGLE (square), LRE, LRLGLE (exponential), SRLGLE (HHL) GLSZM: GLNU, LGZE, SALGLE (all HHH)	**Thyroid Cartilage Invasion**: AUC (LR): 0.876 (95% CI: 0.830–0.913) AUC (LR + SVMSMOTE): 0.905 (95% CI: 0.863–0.937) AUC (Radiologist): 0.721 (95% CI: 0.663–0.774)	Retrospective, single-centre study. Manual slice-by-slice tumour delineation (time-consuming). Only venous phase contrast-enhanced CT images analysed; other phases not compared. CT scans acquired using three different scanners with varying parameters. Radiologist assessments performed by junior radiologists. No external validation performed.	CT radiomics-based models demonstrated high accuracy in predicting thyroid cartilage invasion, outperforming radiologist assessment and showing potential to aid diagnostic decision-making.
**Rao et al. [8]** **2023** **Staging and Histopathology**	To assess whether CT radiomic features (pre-biopsy) can classify T-stage and histological grade in supraglottic laryngeal tumours.	Retrospective single-institution cohort with internal validation using a train–test division.	Contrast-enhanced CT	Pyradiomics	Yes	20	n = 20	Not applicable (Diagnostic)	Internal validation: 80/20 train–test split (n = 16/4)	**Feature selection**: mRMR algorithm used to rank features by relevance and redundancy. **Signature construction**: Selected features used to train SVM models for T-stage (T1–T2 vs. T3–T4) and grade (Grade II vs. Grade III).	**Staging (T1–T2 vs. T3–T4)**: Shape: Max3D, Max2D (row), Flatness FO: Energy, Skewness GLCM: Contrast, DiffAvg, DiffEntropy, Id, Idm, ClusterShade GLRLM: GLNU, LRE, LRHGE GLSZM: LAHGLE NGTDM: Contrast **Grading (Grade II vs. Grade III)**: GLRLM: RLNUN, RP, RV, SRE GLSZM: LAE, LALGLE, ZP, ZV GLDM: DNU, GLNU NGTDM: Busyness, Coarseness, Complexity GLCM: ClusterShade	**T-stage Classification (Low vs. High)**: Accuracy: 74.5% (Training), 69.5% (Validation) AUC (Training): 0.788 (95% CI: 0.698–0.862) AUC (Validation): 0.787 (95% CI: 0.634–0.898) Threshold: 0.49 (Youden Index) Grade Classification (Grade II vs. Grade III): Accuracy: 74.5% (Training), 69.5% (Validation)	Retrospective, single-centre study. Small sample size (<50 laryngeal cancer patients). Manual tumour segmentation by single radiologist. High dimensional feature space relative to sample size. Potential variability in CT imaging protocols not fully addressed. No external validation performed.	CT radiomic features demonstrated moderate discriminatory performance for both T-stage and histological grade classification in supraglottic laryngeal cancer, suggesting potential for non-invasive diagnostic stratification. Validation is necessary on larger data sets. ^1^

^1^ Abbreviations: CT: computed tomography; ICC: intraclass correlation coefficient; LASSO: least absolute shrinkage and selection operator; SVM: support vector machine; SVM-SMOTE: support vector machine—synthetic minority over-sampling technique; GLCM: grey-level co-occurrence matrix; GLRLM: grey-level run length matrix; GLSZM: grey-level size zone matrix; NGTDM: neighbourhood grey tone difference matrix; GLDM: grey-level dependence matrix; FO: first order; mRMR: minimum redundancy maximum relevance; AUC: area under the curve; CI: confidence interval.

**Table 2:** Summary of studies evaluating CT and PET radiomics prognosis in laryngeal cancer.

Author	Study Aim	Study Type and Design	Imaging Modality	Radiomics Software Used	Exclusively Laryngeal Cancer Patients	Total Patients (n)	Laryngeal Cancer Patients (n)	Primary Treatment	Model Validation Strategy	Radiomics Feature Selection and Signature Construction	Significant Radiomic Features	Model Performance/Statistical Outcomes	Limitations	Conclusions
***PET Radiomics for Prognosis in Laryngeal Cancer***														
*Bogowicz et al. [11]* *2017* ***Prognosis***	To evaluate the association between post-CRT 18F-FDG PET radiomics and local tumour control in HNSCC, and to compare two software implementations.	Retrospective single-institution cohort with internal validation using a train–test division	18F-FDG PET/CT	In-house developed software from MAASTRO and University Hospital Zurich	No	178	n = 11	CRT	Internal validation: 70/30 train–test split (n = 128/50)	**Feature selection**: ICC > 0.8 for reproducibility; dimensionality reduction via PCA **Signature construction**: LASSO regression used to construct final model	**Local Control/Recurrence**: Histogram: Range (USZ) GLCM: Difference Entropy (MAASTRO)	**Local Control (Radiomic Models—MAASTRO and USZ)**: C-index (Training): 0.75–0.76 C-index (Validation): 0.71–0.73 Calibration slope (Validation): 1.02–1.13 (well calibrated)	Retrospective, single-centre study. Heterogeneous population including both laryngeal and hypopharyngeal cancers (<50 laryngeal cancer patients). Tumour delineation differences between implementations impacted feature extraction. No external validation performed	An increased histogram range and elevated GLCM difference entropy are associated with a higher risk of tumour recurrence. Both post-treatment PET-CT radiomic models demonstrated prognostic value for local tumour control and showed comparable performance.
*Feliciani et al. [12]* *2018* ***Prognosis***	To assess the value of pre-treatment 18F-FDG PET texture analysis in predicting treatment failure in primary HNSCC treated with concurrent CRT.	Retrospective single-institution cohort with internal validation using cross-validation	18F-FDG PET/CT	CGITA v1.3	No	90	n = 14	CRT	Internal validation: 10-fold cross-validation	**Feature selection**: LASSO regression (1000 iterations); features selected based on occurrence >500/1000 runs **Signature construction**: Multivariate Cox regression using top imaging and clinical features; performance evaluated with Harrell’s C-index and 10-fold cross-validation; Kaplan–Meier used for stratification analysis	**Local Failure**: GLRLM: LILRE	**Local Failure:** **C-index**: 0.76 (radiomics model) vs. 0.65 (clinical model) Multivariate Cox Model: LILRE independently predictive (*p* = 0.001)	Retrospective, single-centre study. Small sample size (<50 laryngeal cancer patients). Heterogeneous patient population and disease sites. Potential treatment selection bias. HPV status not determined for all patients and not included as a predictive factor. No external validation performed.	LILRE from pre-treatment 18F-FDG PET/CT independently predicts local failure in HNSCC patients undergoing CRT. Incorporating texture analysis with clinical variables may improve local control prediction.
*Guezennec* *et al. [13]* *2018* ***Prognosis***	To assess the prognostic value of texture features from 18F-FDG PET/CT in a large cohort of HNSCC patients across all subsites and stages.	Retrospective single-institution cohort with internal validation using cross-validation	18F-FDG PET/CT	LIFEx	No	284	n = 32	Mixed Modalities	Internal validation: 30-fold cross-validation	**Feature selection**: Pearson correlation clustering (r > 0.8); representative features selected based on prior evidence and statistical relevance **Signature construction**: Final features (e.g., SUVmax, MTV, texture) chosen via univariate significance and multivariate relevance	**Overall Survival**: MTV, GLCM: Correlation	**Overall Survival**: Multivariate Cox regression: Treatment (HR = 2.01, 95% CI: 1.29–3.13, *p* = 0.002) MTV (HR = 1.012, 95% CI: 1.003–1.021, *p* = 0.008) GLCM Correlation (HR = 4.51, 95% CI: 1.18–17.24, *p* = 0.02) AUC performance: MTV: AUC = 0.68 (95% CI: 0.62–0.74) GLCM Correlation: AUC = 0.66 (95% CI: 0.60–0.72)	Retrospective, single-centre study. Heterogeneous population including multiple head and neck cancer subsites, not exclusively laryngeal cancer (<50 laryngeal cancer patients). Textural indices not calculable in 26% of patients due to small lesion volume. Use of fixed 40% SUVmax threshold for lesion segmentation, which may require manual adjustment in heterogeneous tumours. No consensus on segmentation. resampling, or calculation parameters for texture analysis applied. No external validation performed.	MTV and GLCM correlation derived from pre-treatment 18F-FDG PET/CT were independent prognostic factors for overall survival in patients with head and neck squamous cell carcinoma.
*Choi et al. [15]* *2023* ***Prognosis***	To evaluate whether combining pre- and post-treatment 18F-FDG PET/CT radiomics with clinical data improves prognostic accuracy in laryngeal and hypopharyngeal cancer.	Retrospective single-institution cohort with internal validation using a train–test division	18F-FDG PET/CT	PET Edge (MIM v7.1.7) for VOI segmentation; CGITA toolbox (via MATLAB 2012a) for radiomic feature extraction, following IBSI guidelines.	No	91	n = 57	CRT	Internal validation: 70/30 train–test split (n = 61/30; includes hypopharyngeal cancer)	**Feature selection**: LASSO regression with n-fold cross-validation used to select PET features associated with PFS/OS **Signature construction**: Delta pre/post radiomic features used to compute Rad-score from non-zero LASSO coefficients; patients stratified via X-tile analysis	**Progression-Free Survival**: Co-occurrence: Normalized Second Angular Moment, Correlation SUV Statistics: SUV Variance **Overall Survival**: Co-occurrence: Contrast SUV Statistics: SUV Variance, SUV Kurtosis, SUV Bias-Corrected Skewness Texture Feature Coding: Co-occurrence Second Angular Moment	**Progression-Free Survival**: Rad-score: HR = 2.15, 95% CI [1.10–4.21], *p* = 0.025 Combined model: C-index = 0.802–0.889 **Overall Survival**: Rad-score: HR = 33.89, 95% CI [2.89–397.18], *p* = 0.005 Combined model: C-index = 0.860–0.958	Retrospective, single-centre study. Small sample size. Heterogeneous population including multiple head and neck cancer subsites, not exclusively laryngeal cancer. Lack of scanner harmonization. Software not fully IBSI-compliant. Potential influence of pre-treatment imaging on treatment decisions. No external validation performed.	Combining delta radiomic features from pre- and post-treatment 18F-FDG PET/CT with clinical data significantly improved prognostic performance for both progression-free and overall survival in laryngeal and hypopharyngeal cancer.
*Nakajo et al. [16]* *2023* ***Prognosis***	To assess whether 18F-FDG PET/CT radiomic features can predict survival and disease progression in laryngeal cancer.	Retrospective single-institution cohort with internal validation using a train–test division	18F-FDG PET/CT	LIFEx	Yes	49	n = 49 (T1–2: 20, T3–4: 29)	Mixed Modalities	Internal validation: 70/30 train–test split (n = 34/15)	**Feature selection**: Gini impurity ranking of radiomic and clinical features; top 10 subsets (5–47 features) evaluated **Signature construction**: Stratified 10-fold cross-validation; CPH and RSF models selected by peak C-index	**Disease Progression**: GLCM: Entropy GLZLM: ZLNU **Progression-Free Survival**: GLCM: Entropy GLZLM: ZLNU GLRLM: LRHGE, SRHGE	**Disease Progression**: AUC (Training): 0.805 AUC (Testing): 0.842 **Progression-Free Survival**: C-index (Training): 0.840 C-index (Testing): 0.808	Retrospective, single-centre study. Small sample size (<50 laryngeal cancer patients). Mixed treatment methods may affect analysis. Radiomic features correlated with tumour size, potentially confounding results. Variable follow-up durations. No external validation performed.	Machine learning models using 18F-FDG PET radiomic and clinical features demonstrated strong predictive performance for disease progression and progression-free survival in laryngeal cancer patients.
*Bogowicz et al. [11]* *2017* ***Prognosis***	To investigate the value of pre-treatment 18F-FDG PET radiomics in modelling local tumour control.	Retrospective single-institution cohort with internal validation using a train–test division	18F-FDG PET/CT and Contrast-enhanced CT	In-house developed software (Python-based)	No	172	n = 10	CRT	Internal validation: 70/30 train–test split (n = 121/51)	**Feature selection**: PCA applied to 569 CT and 18F-FDG PET/CT features **Signature construction**: Separate multivariable Cox models (CT, PET, PET/CT) built with backward selection	**Local Control**: CT—GLSZM: Size Zone Entropy PET—GLSZM: Small ZoneLow Grey-Level Emphasis (SZLGE)	**Local Control**: C-index (Training): CT Model = 0.72, PET Model = 0.74, PET-CT Combined Model = 0.77 C-index (Validation): CT Model = 0.73, PET Model = 0.71, PET-CT Combined Model = 0.73	Retrospective, single-centre study. Limited sample size (<50 laryngeal cancer patients). Heterogeneous population including both laryngeal and hypopharyngeal cancers. GTV delineation subject to inter-observer variability. Autosegmentation may miss metabolically inactive tumour regions. Potential bias from temporal differences between CT and PET acquisitions. Clinical factors such as smoking status and performance status not integrated with radiomic analysis. No external validation performed.	Tumours exhibiting more homogeneous CT density and concentrated areas of high FDG uptake were associated with better prognosis. Radiomic analyses from both CT and PET demonstrated similarly strong ability to discriminate local tumour control in HNSCC.
*Vallieres et al. [14]* *2017* ***Prognosis***	Radiomics combined with clinical data improves prediction of recurrence risk in head and neck cancer. Specific texture features from PET/CT are associated with locoregional recurrence and distant metastases, demonstrating good predictive performance in external validation cohorts.	Retrospective multicentre cohort divided into training and external validation cohorts	18F-FDG PET/CT and Contrast-enhanced CT	In-house developed software (MATLAB-based)	No	300	n = 45	CRT	Internal and external validation: Training cohort (n = 194); external validation on independent cohort (n = 106)	**Feature selection**: Imbalance-adjusted logistic regression with bootstrapping **Signature construction**: Radiomic and clinical features combined using imbalance-adjusted random forest with stratified subsampling validation	**Locoregional Recurrence**: GLSZM: LZHGE **Distant Metastases**: GLSZM: ZSN **Overall Survival**: GLRLM: GLV	**Locoregional Recurrence**: AUC (Validation): 0.69 C-index (Validation): 0.67 **Distant Metastasis**: AUC (Validation): 0.86 C-index (Validation): 0.88	Retrospective study. Heterogeneous population including multiple head and neck cancer subsites, not exclusively laryngeal cancer (<50 laryngeal cancer patients). Inter-observer variability in tumour delineation not fully addressed. Key clinical factors like smoking and performance status excluded. Variability from imaging protocols and feature extraction needs standardisation	Radiomics offers valuable prognostic insights for locoregional recurrence and distant metastases in head and neck cancer.
***CT Radiomics for Prognosis in Laryngeal Cancer***														
*Zhang et al. [9]* *2013* ***Prognosis***	To evaluate the association between CT radiomics and overall survival in locally advanced HNSCC patients treated with induction chemotherapy.	Retrospective single-institution study; no internal or external validation.	Contrast-enhanced CT	TexRad	No	72	n = 21	Induction Chemotherapy ± Definitive Treatment	No formal validation: Multivariate Cox regression	**Feature selection**: No formal selection; predefined texture (entropy) and histogram features extracted using TexRAD at multiple spatial scales. **Signature construction**: Cox proportional hazards models used to assess associations between imaging features and overall survival, adjusted for clinical covariates.	**Overall Survival**: First order: Entropy, Skewness	**Overall Survival** (Multivariate Cox Regression): Tumour size: HR = 1.58, *p* = 0.018 N stage (N3 vs. N0/N1): HR = 8.77, *p* = 0.002 N stage (N3 vs. N2): HR = 4.99, *p* = 0.001 Entropy: HR = 2.10, *p* = 0.036 Skewness: HR = 3.67, *p* = 0.009	Retrospective, single-centre study. Small sample size (<50 laryngeal cancer patients). Heterogeneous population including multiple head and neck cancer subsites, not exclusively laryngeal cancer. Single-user semi-automated segmentation; interobserver variability not assessed. Unclear reproducibility of CT texture parameters across institutions and different scanning protocols. Clinical and pathological treatment response not incorporated into analysis. No internal or external validation performed.	CT radiomic features, specifically entropy and skewness, alongside clinical factors including tumour size and nodal stage, were independently associated with overall survival in locally advanced HNSCC patients treated with induction chemotherapy. No formal validation was performed.
*Ou et al. [10]* *2017* ***Prognosis***	To evaluate the prognostic value of radiomics in locally advanced HNSCC treated with CRT or BRT.	Retrospective single-institution cohort with internal validation using cross-validation.	Contrast-enhanced CT	Oncoradiomics (MATLAB-based)	No	120	n = 18	CRT or bioradiotherapy	Internal validation: 10-fold cross-validation	**Feature selection**: 24 radiomic features selected after FDR correction; dimensionality reduced using PCA. **Signature construction**: Radiomics signature developed from first principal component (PC1); optimal cutoff defined using Youden index on ROC for 5-year OS.	**Overall Survival and Progression-Free Survival**: Shape: MaxDiameter2Dy, MaxDiameter2Dz, MaxDiameter3D, Sphericity Disproportion, Surface, Volume FO: Range, Total Energy, Min (HLL), Energy (HHL), Total Energy (HHL, LLL) GLCM (HLL): Autocorrelation, Sum Average, Sum Squares, Sum Variance GLSZM: High Intensity Large Area Emphasis; High Intensity Small Area Emphasis (HHL, HLL); High Intensity Emphasis (HLL); High Intensity Large Area Emphasis (LLL) GLRLM (HLL): HGRE, LRHGE, SRHGE	**Overall Survival**: HR = 0.30, *p* = 0.02 **Progression-Free Survival**: HR = 0.30, *p* = 0.01 5-year AUC: Combined (Radiomics + p16): 0.78 p16 alone: 0.64 (*p* = 0.01) Radiomics alone: 0.67 (*p* = 0.01) **Additional Findings**: Patients with high radiomic scores benefited significantly from CRT over BRT (*p* = 0.004) Signature with p16 stratification showed significant OS/PFS differences (*p* < 0.001)	Retrospective, single-centre study. Small sample size (<50 laryngeal cancer patients). Heterogeneous population including multiple head and neck cancer subsites, not exclusively laryngeal cancer. Variability in CT acquisition protocols over study period. HPV status determined by p16; IHC only without confirmatory testing. No external validation performed.	A radiomics signature derived from CT features significantly predicted overall and progression-free survival in locally advanced HNSCC treated with CRT or BRT. Combining radiomics with p16 status improved prognostic accuracy, and patients with high radiomic scores showed greater benefit from CRT over BRT.
*Kuno et al. [21]* *2017* ***Prognosis***	To assess the prognostic value of radiomic texture features from pre-treatment CT in HNSCC patients treated with CRT.	Retrospective single-institution study; no internal or external validation.	Contrast-enhanced CT	In-house developed software (MATLAB-based)	No	62	n = 19	CRT	No formal validation: Univariate and multivariate Cox regression	**Feature selection**: Texture features associated with local failure identified via Mann–Whitney U test. **Signature construction**: Univariate and multivariate Cox regression models used; ROC analysis applied to define optimal thresholds.	**Local Failure**: Histogram: Geometric Mean, Harmonic Mean, Fourth Moment GLRLM: SRE, GLN, RLN, SRLGLE	**Local Failure**: Multivariate Cox model predictors: Histogram: Geometric Mean (HR = 4.68, *p* = 0.026), Harmonic Mean (HR = 8.61, *p* = 0.004), Fourth Moment (HR = 4.56, *p* = 0.048) GLRLM: SRE (HR = 3.75, *p* = 0.044), GLN (HR = 5.72, *p* = 0.004), RLN (HR = 4.15, *p* = 0.043), SRLGE (HR = 5.94, *p* = 0.035) ROC performance: AUC (RLN): 0.82 Sensitivity: 77.3% Specificity: 77.5%	Retrospective, single-centre study. Small sample size (<50 laryngeal cancer patients). Heterogeneous population including multiple head and neck cancer subsites, not exclusively laryngeal cancer. Non-uniform CT protocols (scanner types, section thickness, reconstruction) potentially affecting texture analysis. Single-user semiautomated segmentation. interobserver variability not assessed. Exclusion of necrotic/ulcerated tumour areas from analysis, which may impact texture features related to hypoxia/radiosensitivity. No internal or external validation performed.	Pre-treatment CT radiomic texture features independently predicted local failure in HNSCC. Key predictors included histogram and GLRLM metrics. Findings highlight potential for non-invasive risk stratification, though no external validation was conducted.
*Chen et al.* [22] *2020* ***Prognosis***	To evaluate the prognostic value of a CT-based radiomics signature and nomogram in patients with laryngeal cancer following surgical resection.	Retrospective single-institution cohort with internal validation using a train–test division.	Contrast-enhanced CT	LIFEx	Yes	136	n = 136 (T1–2: 83, T3–T4: 53)	Surgery ± adjuvant therapy	Internal validation: Train–test split (n = 96/40)	**Feature selection**: Stable features retained using ICC > 0.8; LASSO Cox regression used to select six features with non-zero coefficients. **Signature construction**: Rad-score calculated from selected features; integrated with clinical variables into a prognostic nomogram.	**Overall Survival**: GLRLM: HGRE, LRHGE GLZLM: ZLNU	**Overall Survival**: Radiomics Signature: C-index (Training): 0.782 (95% CI: 0.656–0.909, *p* = 0.170) C-index (Validation): 0.752 (95% CI: 0.614–0.891, *p* = 0.456) Clinical Nomogram: C-index (Training): 0.802 (95% CI: 0.690–0.914, *p* = 0.007) C-index (Validation): 0.807 (95% CI: 0.630–0.985, *p* = 0.192) Radiomics Nomogram: C-index (Training): 0.817 (95% CI: 0.693–0.942, *p* = 0.009) C-index (Validation): 0.913 (95% CI: 0.833–0.992, *p* = 0.019) AJCC Staging System: C-index (Training): 0.682 (95% CI: 0.553–0.812) C-index (Validation): 0.699 (95% CI: 0.458–0.941)	Retrospective, single-centre study. Small sample size. Variability in tumour delineation despite use of computer-aided software. Potential confounding from variations in therapeutic strategies and complications. Lack of standardisation in texture feature extraction and image processing software. No external validation performed.	Integrating radiomic features into a prognostic nomogram significantly improved overall survival prediction accuracy in laryngeal cancer patients post-surgery, outperforming both clinical-only models and AJCC staging.
*Agarwal et al. [19]* *2020* ***Prognosis***	To determine whether pre-treatment CT texture features can predict long-term local control and laryngectomy-free survival in locally advanced laryngopharyngeal carcinoma.	Retrospective single-institution cohort with internal validation using cross-validation..	Contrast-enhanced CT	Pyradiomics	No	60	n = 31	CRT	Internal validation: 10-fold cross-validation	**Feature selection**: ANOVA F-test followed by LASSO regression. **Signature construction**: Random forest and logistic regression models trained on selected features.	**Laryngectomy-Free Survival**: FO (medium texture): Entropy, Kurtosis, Skewness, Standard Deviation **Local Control**: FO (medium texture): Entropy, Skewness	**Laryngectomy-Free Survival**: Medium filter Entropy ≥ 4.54: *p* = 0.006 Kurtosis ≥ 4.18: *p* = 0.019 Skewness ≤ −0.59: *p* = 0.001 Standard deviation ≥ 43.18: *p* = 0.009 Independent predictor: Medium filter Entropy (*p* < 0.001) **Local Control**: Medium filter Entropy ≥ 4.54: *p* = 0.01 Skewness ≤ −0.59: *p* = 0.02 Fine filter Entropy ≥ 4.29 and Kurtosis ≥ −0.27: *p* = 0.01 (both) Independent predictor: Medium filter Entropy (*p* = 0.001)	Retrospective, single-centre study. Small sample size (<50 laryngeal cancer patients). Heterogeneous population including multiple head and neck cancer subsites, not exclusively laryngeal cancer. Tumour delineation performed by a single operator. Single-slice tumour delineation instead of full volumetric analysis. No external validation performed.	Medium-filtered CT texture features, particularly entropy, were significant independent predictors of both local control and laryngectomy-free survival in patients with locally advanced laryngopharyngeal carcinoma.
*Keek et al. [23]* *2020* ***Prognosis***	To investigate whether CT radiomics of peritumoural tissue can predict overall survival, locoregional recurrence, and distant metastases in advanced HNSCC treated with CRT.	Retrospective multicentre cohort combining DESIGN and BD2Decide datasets with internal validation using a train–test division.	Contrast-enhanced CT	RadiomiX Discovery Toolbox (Oncoradiomics)	No	444	n = 57	CRT	Internal validation: 100-repeat 2-fold cross-validation	**Feature selection**: Univariate Cox regression (FDR-adjusted *p* < 0.05); features retained if selected in >50% of 100 CV runs. **Signature construction**: Multivariate Cox and RSF models using top-ranked features; clinical and radiomic models developed separately and compared.	No features reached statistical significance	**Clinical Model (Best Performance)**: C-index (Validation): 0.75 (Cox), 0.65 (RSF) **Peritumoral Radiomics Models**: C-index (Validation): 0.32–0.61	Retrospective study. Heterogeneous population including multiple head and neck cancer subsites, not exclusively laryngeal cancer. Differences in clinical variables between training and validation sets. Radiomics focused on peritumoral regions with limited volume affecting feature extraction reliability. Potential biological complexity of recurrence limits predictive power of bulk tumour radiomics. No external validation performed.	Radiomic features from the peritumoral regions are not useful for the prediction of time to OS, LR, and DM.
*Meneghetti et al. [20]* *2020* ***Prognosis***	To develop and validate a CT-based radiomics signature for predicting locoregional control in HNSCC patients treated with primary CRT.	Retrospective multicentre cohort with internal cross-validation and external validation.	Contrast-enhanced CT	MIRP (Medical Imaging Radiomics Processor), Python-based	No	318	n = 8	CRT	Internal and external validation: 3-fold cross-validation (n = 233); external validation on independent cohort (n = 85)	**Feature selection**: ICC filtering, Spearman, MRMR, and LASSO across repeated 3-fold CV. **Signature construction**: Cox, boosted Cox, and RSF models; final signature selected by highest median C-index.	**Local Control**: Shape: GTV First-order: stat_p10 Texture (GLDM, log): High Dependence High Grey-Level Emphasis	**Clinical Model (GTV-only)**: C-index (Training): 0.59 (95% CI: 0.53–0.65) C-index (Validation): 0.61 (95% CI: 0.51–0.71) **Clinical–Radiomics Model**: C-index (Training): 0.63 (95% CI: 0.58–0.69) C-index (Validation): 0.66 (95% CI: 0.55–0.75)	Retrospective study. Heterogeneous population including multiple head and neck cancer subsites, not exclusively laryngeal cancer (<50 laryngeal cancer patients). Moderate sample size although very small number of laryngeal cancer patients. Underreporting of some model details as per TRIPOD recommendations. Complex modelling approach may limit reproducibility in other settings. Limited prospective validation; external validation cohort relatively small.	The final signature combined tumour volume with two independent radiomic features, achieving moderate discriminatory performance for predicting locoregional control in a validation cohort.
*Kang et al. [17]* *2023* ***Prognosis***	To develop a radiomics nomogram for predicting pathological response and overall survival after induction chemotherapy in advanced laryngeal cancer.	Retrospective single-institution cohort with internal validation using a train–test division.	Contrast-enhanced CT	3D-Slicer	Yes	114	n = 114 (T1–2: 0, T3–4: 114)	Mixed Modalities	Internal validation: 70/30 train–test split (n = 81/33)	**Feature selection**: Stable features (ICC > 0.8) selected; LASSO regression with 100 iterations of 10-fold CV applied. **Signature construction**: Rad-score computed from selected features; patients stratified by median score; nomogram built with radiomic and clinical features.	**Overall Survival**: GLSZM: SizeZoneNonUniformity GLCM: ClusterProminence GLDM (LHH): LargeDependenceEmphasis GLDM (HHL): LargeDependenceEmphasis GLDM: LargeDependenceEmphasis NGTDM: Complexity	**Overall Survival (1-year)**: AUC (Training): 0.802 AUC (Validation): 0.735 **Overall Survival (3-year)**: AUC (Training): 0.789 AUC (Validation): 0.746	Retrospective, single-centre study. Limited sample size. Mixed treatment methods may affect analysis. No integration of gene transcriptome data. No external validation performed.	Radiomics-enhanced nomogram moderately improved overall survival prediction in advanced laryngeal cancer, demonstrating potential for non-invasive, individualized treatment planning.
*Woolen et al. [18]* *2021* ***Prognosis***	To evaluate whether CT perfusion and radiomic features from pre- and post-treatment imaging can predict one-year disease-free survival in laryngeal and hypopharyngeal cancer.	Retrospective secondary analysis of a phase II trial with internal validation via cross-validation.	Contrast-enhanced CT and CT perfusion	In-house developed software	No	44	n = 42 (T1–2: 0, T3–4: 42)	Induction Chemotherapy ± Definitive Treatment	Internal validation: Two-loop leave-one-out cross-validation	**Feature selection**: Two-loop leave-one-out cross-validation. **Signature construction**: Linear discriminant analysis classifier used to build a combined response index from selected radiomic, perfusion, and laryngoscopic features.	**Disease-Free Survival**: Change in blood flow, Percent change in tumour volume (pre- vs. post-therapy), delta radiomics	**Disease Progression**: AUC (Training): 0.68 (95% CI: 0.50–0.85) AUC (Validation): 0.69 (95% CI: 0.50–0.85) **Laryngoscopic Assessment**: AUC (Validation): 0.40	Retrospective, single-centre study. Small sample size (<50 laryngeal cancer patients). Heterogeneous population including both laryngeal and hypopharyngeal cancers. Semi-autonomous segmentation with potential inter- and intra-observer variability, though not statistically significant. Limited evaluation of imaging protocol variability. No external validation performed	Combined CT perfusion and radiomic features modestly improved prediction of one-year disease-free survival compared to laryngoscopic assessment.
*Cozzi et al. [24]* *2019* ***Prognosis***	To evaluate whether a CT-based radiomics signature can predict clinical outcomes following CRT in stage III–IV HNSCC.	Retrospective single-institution cohort with internal validation using a train–test division.	Non-contrast CT	LIFEx	No	110	n = 11	CRT	Internal validation: Train–test split (n = 70/40)	**Feature selection**: Univariate analysis (FDR < 0.2) followed by Elastic Net regularisation. **Signature construction**: Multivariate Cox regression used to build Rad-score; patients stratified by median value.	**Overall Survival**: GLRLM: RLNU GLZLM: GLNU NGLDM: Coarseness **Progression-Free Survival**: Shape: Compacity GLCM: Correlation **Local Control**: Shape: Volume NGLDM: Coarseness	**Overall Survival**: C-index (Training): 0.88 C-index (Validation): 0.90 **Progression-Free Survival**: C-index (Training): 0.72 C-index (Validation): 0.80 **Local Control**: C-index (Training): 0.72 C-index (Validation): 0.82	Retrospective, single-centre study. Limited sample size (<50 laryngeal cancer patients). Heterogeneous population including multiple head and neck cancer subsites, not exclusively laryngeal cancer. Single-expert manual segmentation without assessment of inter-observer variability. Differing CT acquisition parameters and resampling methods on feature extraction. Feature selection methods may overlook complex feature interactions. Did not include higher-order or wavelet features. Absence of artifact correction and test–retest stability assessment. Use of simple statistical models; different machine learning methods might yield different results. No external validation performed.	CT-based radiomics signature demonstrates strong predictive ability for overall survival, progression-free survival, and local control in advanced HNSCC patients treated with CRT. This non-invasive tool may enhance risk stratification and support personalized treatment planning. ^1^

^1^ Abbreviations: AUC: area under the curve; BRT: bioradiotherapy; CI: confidence interval; CPH: Cox proportional hazards; CRT: chemoradiotherapy; CT: computed tomography; DFS: disease-free survival; FO: first order; FDG: fluorodeoxyglucose; GLCM: grey-level co-occurrence matrix; GLDM: grey-level dependence matrix; GLRLM: grey-level run-length matrix; GLSZM: grey-level size zone matrix; GTV: gross tumour volume; HNSCC: head and neck squamous cell carcinoma; HR: hazard ratio; ICC: intraclass correlation coefficient; IHC: immunohistochemistry; LASSO: least absolute shrinkage and selection operator; LC: local control; LFS: laryngectomy-free survival; MTV: metabolic tumour volume; NGTDM: neighbouring grey-tone difference matrix; OS: overall survival; PCA: principal component analysis; PET: positron emission tomography; PFS: progression-free survival; Rad-score: radiomic score; ROC: receiver operating characteristic; RSF: random survival forest; SUV: standardised uptake value; VOI: Volume of interest; ZLNU: zone length non-uniformity.

### 3.5. Methodological Quality Assessment (Radiomics Quality Score)

Methodological quality of the included studies was formally assessed using RQS (version 1.0), with results summarised in Figure 3. Total RQS scores ranged from 0% to 64% of the maximum possible score, indicating substantial variability in methodological rigour across the literature. The majority of studies achieved low to moderate scores, indicating that methodological limitations remain common and that many radiomics models are not yet ready for clinical translation.

Assessment across individual RQS domains revealed consistent patterns. Most studies reported imaging acquisition parameters and applied feature reduction or multiple testing strategies. In contrast, key methodological limitations were frequently observed, including absence of test–retest or phantom analyses, limited use of independent external validation cohorts, and a lack of formal clinical utility or decision-impact analyses. Higher RQS scores were typically achieved by studies incorporating external validation and more comprehensive statistical reporting, whereas studies without validation or robustness assessment generally demonstrated lower overall scores.

## 4. Discussion

### 4.1. Synthesis of Key Findings

This review synthesised 20 radiomic studies focusing on laryngeal cancer or mixed cohorts containing laryngeal subsites, grouped according to modelling objective. Across staging, survival prediction, recurrence modelling, and treatment response, radiomics has consistently shown potential to extract prognostically relevant imaging features from standard-of-care CT and PET/CT scans.

Several radiomic features were repeatedly identified as predictive across studies. Entropy and skewness, both first-order features reflecting intensity distribution and heterogeneity, were among the most frequently selected [6,7,8,11,15,16,26]. Second-order features derived from GLCM and GLSZM matrices also emerged consistently, irrespective of imaging modality or disease endpoint [6,7,8,10,11,13,14,16,17,24,25]. This cross-study recurrence suggests a potential underlying biological relevance, possibly reflecting tumour complexity, aggressiveness, and response to therapy. Higher entropy and skewness are likely to reflect increased intratumoural heterogeneity, necrosis, and hypoxic burden, biological features that have been consistently associated with aggressive tumour behaviour, treatment resistance, and poorer prognosis in laryngeal squamous cell carcinoma.

Clinically, the consistent selection of certain features across modalities and endpoints supports their potential as imaging biomarkers. Risk stratification using radiomics signatures could assist with decisions around treatment intensification, organ preservation, or surveillance intensity, provided these tools are interpretable, externally validated, and embedded in clinical workflows.

### 4.2. Strengths and Limitations of the Current Evidence

Formal assessment using the RQS provides an objective framework for interpreting the strengths and limitations of the current evidence base. As demonstrated in Figure 3, methodological quality varied widely across studies, with most achieving low to moderate RQS scores. Higher scores were observed in a small number of studies, typically characterised by more comprehensive reporting of imaging protocols, robust feature reduction strategies, and incorporation of independent external validation cohorts.

Several strengths are evident in the evolving literature. Methodologically, more recent studies, such as those included in the review, demonstrate increasing rigour. The use of machine learning classifiers, such as random forests, SVMs, and LASSO, reflects a growing sophistication in modelling. Importantly, some studies now incorporate delta-radiomics [15,18], tracking feature evolution across treatment timepoints; a promising direction for predicting response and adapting therapy [27].

Additionally, performance evaluation has matured. Some studies reported not only AUCs or C-indices but also calibration [11], risk stratification thresholds [15,21,24], and, in a few cases, external validation [14,20]. Several adopted consensus segmentation approaches, and a handful followed IBSI guidelines [5].

Despite this progress, significant limitations remain. Cohort size is a persistent issue. Only 6/20 studies focused exclusively on laryngeal cancer [6,7,8,16,17,22], and many involved fewer than 50 laryngeal patients [8,10,11,12,14,16,18,20,21,24,25,26]. Small sample sizes limit statistical power and increase the risk of overfitting, particularly in high-dimensional radiomic feature spaces [28]. Furthermore, the use of mixed head and neck cancer cohorts without laryngeal-specific subgroup analysis introduces biological and clinical heterogeneity, as tumour behaviour, treatment decisions, and prognostic implications vary significantly between subsites [29]. Importantly, these associations likely reflect fundamental differences in tumour biology across head and neck subsites. Laryngeal squamous cell carcinoma is predominantly smoking-related, whereas oropharyngeal cancers are frequently virally driven (HPV-associated) and nasopharyngeal cancers are commonly linked to Epstein–Barr virus infection. These distinct carcinogenic pathways are associated with differences in tumour microenvironment, cellular heterogeneity, angiogenesis, immune infiltration, and growth patterns, which are plausibly captured by radiomic texture and intensity features. These differences in tumour microenvironment and growth patterns may underlie observed associations between radiomic heterogeneity metrics and adverse clinical outcomes. As such, radiomic signatures derived from laryngeal tumours should not be assumed to generalise across virally driven head and neck cancers, reinforcing the need for subsite-specific model development and validation [29].

There is also notably variability in image acquisition and segmentation. Differences in scanner type, reconstruction parameters, and contrast use can affect reproducibility. While some studies addressed this with resampling or harmonisation strategies, few applied techniques such as ComBat [30] outperformed phantom calibration [31]. Manual segmentation, often performed by a single observer, was the norm, with limited assessment of inter-observer variability or reproducibility.

Most studies used internal validation only, such as train–test split or k-fold cross-validation, and external validation was rare [14,20]. None of the studies included prospective validation. This constrains generalisability and inflates reported model performance [32]. Furthermore, several studies failed to report calibration metrics or decision curve analyses, limiting the assessment of clinical utility.

### 4.3. Limitations of This Review

This review employed a structured narrative synthesis, which, while appropriate for addressing methodological and clinical heterogeneity, precluded formal meta-analysis. Although a formal methodological quality assessment was undertaken using the Radiomics Quality Score, this framework does not capture all potential sources of bias, and findings should therefore be interpreted in conjunction with the qualitative appraisal presented. Only English-language publications were included, which may introduce selection bias.

### 4.4. Clinical Applicability and Generalisability

Radiomics holds considerable promise as a non-invasive biomarker in laryngeal cancer, with potential applications in prognosis, treatment selection, and response monitoring. However, as highlighted by the overall RQS assessment, limited external validation, reproducibility testing, and formal clinical utility analyses currently constrain translation into routine clinical practice. Standardisation across the radiomics pipeline remains a foundational requirement. Differences in imaging protocols, segmentation methods, feature extraction, and modelling approaches contribute to inconsistent results and hinder reproducibility. Adoption of consensus frameworks such as those provided by the IBSI [5], along with compliance with reporting guidelines like TRIPOD [33] and CLAIM [34], is essential to improve transparency and reproducibility across studies.

Larger, harmonised, multicentre datasets are needed to evaluate model generalisability and support regulatory progression. Initiatives such as the AIRSPACE project, a mixed retrospective and prospective cohort based across centres in the northern UK, may provide an opportunity to evaluate radiomics models within more standardised imaging pipelines and to support independent validation when fully established. Importantly, such efforts should be viewed as complementary to, rather than a substitute for, rigorous external validation across diverse healthcare settings [3].

## 5. Conclusions

Radiomics has shown promising utility as a non-invasive biomarker in laryngeal cancer, with applications spanning tumour staging, prognostic modelling, and prediction of treatment response. However, widespread clinical adoption remains limited by methodological inconsistencies, small cohort sizes, and lack of standardisation. Future efforts must prioritise robust validation, integration with clinical and molecular data, and alignment with real-world workflows.

## Data Availability

The original data presented in the study are openly available in MEDLINE and EMBASE online databases at https://ovidsp.ovid.com/ (accessed on 5 February 2024).

## Supplementary Materials

The following supporting information can be downloaded at https://www.mdpi.com/article/10.3390/cancers18020237/s1; Supplementary S1: Full electronic search strategy.

## Abbreviations

The following abbreviations are used in this manuscript:

AUC	Area Under Curve
BRT	Bioradiotherapy
CI	Confidence Interval
CLAIM	Checklist for Artificial Intelligence in Medical Imaging
CPH	Coz Proportional Hazards
CRT	Chemoradiotherapy
CT	Computed Tomography
DFS	Disease-Free Survival
FO	First Order
FDG	Fluorodeoxyglucose
GLCM	Grey-Level Co-Occurrence Matrix
GLDM	Grey-Level Dependence Matrix
GLRLM	Grey-Level Run-Length Matrix
GLSZM	Grey-Level Size Zone Matrix
GTV	Gross Tumour Volume
HNSCC	Head and Neck Squamous Cell Carcinoma
HR	Hazard Ratio
IBSI	Image Biomarker Standardisation Initiative
ICC	Intraclass Correlation Coefficient
IHC	Immunohistochemistry
LASSO	Least Absolute Shrinkage and Selection Operator
LC	Local Control
LFS	Laryngectomy-Free Survival
LSCC	Laryngeal Squamous Cell Carcinoma
MRI	Magnetic Resonance Imaging
mRMR	Minimum Redundancy Maximum Relevance
MTV	Metabolic Tumour Volume
NGTDM	Neighbouring Grey-Tone Difference Matrix
OS	Overall Survival
PCA	Principal Component Analysis
PET	Positron Emission Tomography
PFS	Progression-Free Survival
Rad-score	Radiomic score
ROC	Receiver Operating Characteristic
RSF	Random Survival Forest
SUV	Standardised Uptake Value
SVM	Support Vector Machine
SVM-SMOTE	Support Vector Machine—Synthetic Minority Over-sampling Technique
TRIPOD	Transparent Reporting of a multivariable prediction model for Individual Prognosis Or Diagnosis
VOI	Volume of Interest
ZLNU	Zone Length Non-Uniformity
