# Supplementary material for: Radiomics from Routine CT and PET/CT Imaging in Laryngeal Squamous Cell Carcinoma: A Systematic Review with Radiomics Quality Score Assessment
Source: Cancers (Basel). 2026 Jan 13;18(2):237. doi: 10.3390/cancers18020237 (PMC12839367; doi:10.3390/cancers18020237)
Supplement: Supplementary file 1 [file cancers-18-00237-s001.zip › cancers-4023880-supplementary.docx]

**Supplementary S1. Full electronic search strategy**

**Review registration:** PROSPERO (CRD420251117983)

**Searched Databases:**

1. MEDLINE
2. EMBASE

**Platform:** Ovid (Wolters Kluwer)

**Date of search:** May 2025

**Publication date limits:** 1 January 2010 to 31 January 2024

**Language restrictions:** English language only

**Study type restrictions:**

1. Human studies
2. Original research articles only

**MEDLINE and EMBASE (Ovid) search strategy pipeline:**

1. laryngeal cancer.mp.
2. larynx cancer.mp.
3. larynx.mp.
4. laryngeal squamous cell carcinoma.mp.
5. 1 OR 2 OR 3 OR 4
6. radiomics.mp.
7. texture analysis.mp.
8. machine learning.mp.
9. artificial intelligence.mp.
10. 6 OR 7 OR 8 OR 9
11. 5 AND 10
12. Limit 11 to English language
13. Limit 12 to humans
14. Limit 13 to publication year 2010–2024

**Additional search methods:**

Duplicate records were removed prior to screening. Reference lists of included studies and relevant systematic reviews were manually screened to identify additional eligible articles.

**Supplementary S1.** **Full electronic search strategy**

Full electronic search strategy used for identification of studies evaluating radiomic features in relation to laryngeal cancer outcomes. The figure details the MEDLINE and EMBASE search strategies, including search terms, Boolean logic, and applied limits.
